# RNA Phage VLP-Based Vaccine Platforms

**DOI:** 10.3390/ph14080764

**Published:** 2021-08-04

**Authors:** David S. Peabody, Julianne Peabody, Steven B. Bradfute, Bryce Chackerian

**Affiliations:** 1 Department of Molecular Genetics and Microbiology, University of New Mexico School of Medicine, Albuquerque, NM 87131, USA; jpeabody@salud.unm.edu (J.P.); BChackerian@salud.unm.edu (B.C.); 2 Center for Global Health, Division of Infectious Diseases, Department of Internal Medicine, University of New Mexico, Albuquerque, NM 505, USA; sbradfute@salud.unm.edu

**Keywords:** virus-like particle, VLP, RNA bacteriophage, vaccines

## Abstract

Virus-like particles from a variety of RNA bacteriophages have turned out to be useful platforms for delivery of vaccine antigens in a highly immunogenic format. Here we update the current state of development of RNA phage VLPs as platforms for presentation of diverse antigens by genetic, enzymatic, and chemical display methods.

## 1. Introduction

Since their discovery in the early 1960s the single-strand RNA bacteriophages have played important roles in the development of Molecular Biology. Even now, deep in the genomics era it is worth remembering that bacteriophage MS2′s was the first genome ever sequenced [1]. Before recombinant DNA, RNA phages were extensively utilized in studies of protein synthesis because they provided unique access to a pure form of mRNA. They also provided an extensively studied example of RNA-protein recognition, and have served as powerful models of RNA virus structure and assembly. We now know the detailed 3D structures of something such as 30 RNA phage capsids. To better grasp the full scope of knowledge in this area, consult Paul Pumpen’s comprehensive treatise, single-stranded RNA phages: from molecular biology to nanotechnology [2]. Here, we focus on the use of RNA phage-derived virus-like particles as platforms for vaccine development.

The term virus-like particle (VLP) has several alternative usages [3], so we begin by noting that for our purposes a VLP is the non-infectious capsid that forms when viral structural proteins self-assemble in the absence of an intact viral genome. Structurally and immunologically they can be virtually indistinguishable from authentic virions, which explains why VLPs derived from Human papilloma and Hepatitis-B virus structural proteins have been so effective as vaccines against their corresponding viruses. Here we describe the use of VLPs as scaffolds for immunogenic presentation of heterologous antigens. We focus on VLP-based platforms derived from several specific RNA bacteriophages, emphasizing our own experience where it applies, but also describing some other relevant advances in the field.

Why are RNA phage VLPs useful as vaccine platforms? First, like virus particles generally, VLPs are intrinsically immunogenic. Their nano-particulate nature ensures efficient uptake by antigen-presenting cells and their densely repetitive multivalent structures promote efficient B-cell receptor cross-linking, thereby stimulating differentiation and antibody production [4,5,6]. These features combine to guarantee that almost anything presented on the VLP surface elicits a strong antibody response. Furthermore, the ability of these VLPs to package their own mRNAs creates the genotype-phenotype linkage necessary for an affinity-selection technology analogous to phage display. This means that an epitope can be identified by biopanning on a neutralizing antibody target, and then presented to the immune system in the same structural context present during its affinity-optimization. In favorable cases, the resulting VLP selectant can serve as a vaccine to elicit antibodies with specificities similar to that of the selecting antibody.

**Figure 1:**
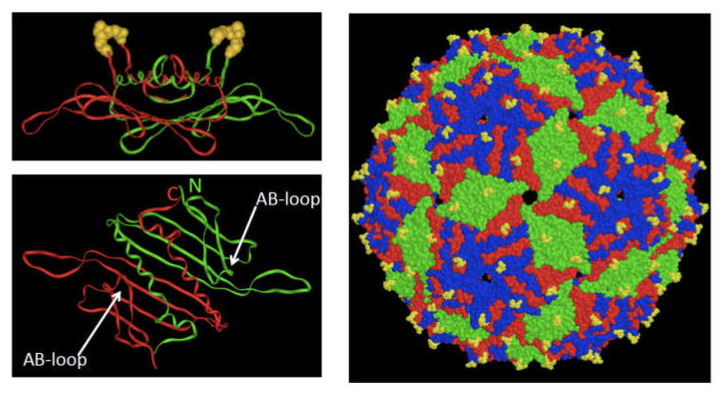
The structure of RNA phage coat proteins and VLPs as exemplified by MS2. At the bottom left is a top-down view of the coat protein dimer. Note the proximity of N- and C-termini of the two subunits, which facilitated construction of a single-chain dimer, and the locations of the AB-loops. At the top left is a side-view of the dimer with its AB-loops in yellow space-fill. On the right, the structure of the VLP showing the density of display of AB-loop surface display (yellow).

For our purposes, bacteriophage MS2 will serve as the prototype. The viral capsid consists of 180 copies of a 129-amino acid coat protein. It folds as a homodimer of intertwining subunits (Figure 1) with dimerization accomplished, in part, by edge-to-edge interactions between each subunit’s 5-stranded ß-sheet [7,8]. Additionally, each monomer throws an alpha-helical arm over the ß-sheet of its companion subunit, thus forming the protein’s hydrophobic core and completing the dimer-stabilizing interactions. The resulting 10-stranded ß-sheet provides the recognition surface for a unique hairpin in viral RNA. This specific RNA-protein interaction mediates coat protein’s translational repression and genome encapsidation functions. The co-assembly of coat protein dimers with one copy of the maturase protein and the 3569 nucleotide RNA genome completes the viral particle. Maturase is essential for virus infectivity because it mediates receptor binding and genome entry, but neither the maturase protein nor the viral genome are necessary for assembly of 90 dimers into the icosahedral VLPs that form when coat protein is expressed from a plasmid in *E. coli*. This makes possible the manipulation of the VLP by conventional recombinant DNA methods in plasmid-based systems entirely divorced from the other requirements of the phage’s biology. The other RNA phages broadly follow the molecular precedents set by MS2. We will note differences as they become relevant.

## 2. The Bacteriophage MS2 VLP as Display Platform

A surface loop connecting MS2 coat protein’s A and B ß-strands is prominently displayed on the VLP surface and offers a natural location for display of foreign peptides (Figure 1). The first use of MS2 for epitope display was reported in 1993 with the construction of several AB-loop insertions [9]. One, for example, displayed a 24-amino-acid peptide from influenza virus, which turned out to be strongly immunogenic in mice. Unsurprisingly however, most AB-loop insertions perturb coat protein folding [10,11]. This initially limited MS2′s utility as a display platform but later construction of a single-chain dimer enhanced the protein’s tolerance of insertions by stabilizing it against all sorts of mutational perturbations [10,11,12]. Figure 1 shows the physical proximity of the N- and C-termini of coat protein subunits in the dimer. Duplicating the coat-encoding DNA sequence and then fusing the reading frames of the two copies created the single-chain dimer. It maintains both the RNA binding and VLP assembly functions of the wild-type in a protein that is far more stable and considerably more tolerant of a variety of mutational insults [10,11,13,14]. Initial experiments showed that random sequence peptides up to ten amino acids (the longest then tested) were tolerated almost universally when inserted into the AB-loop of the single-chain dimer’s C-terminal half. The frequency of folding failures increased with increasing peptide length, but we now know that peptides up to at least 24 amino acids are frequently accepted, especially at lower growth temperatures. Because both ends of AB-loop insertions are tethered, the displayed peptides are conformationally constrained. An alternative, unconstrained display site is present at the coat protein N-terminus. N-terminal fusions tend to be less disruptive of protein folding since they do not interrupt the coat protein sequence, but even they benefit from the stabilizing influence of the single-chain dimer. Furthermore, because it has only half the number of N-termini, use of the single-chain dimer reduces the crowding at three-fold capsid symmetry axes that might sometimes interfere with VLP assembly [10].

The existence of display sites that broadly tolerate peptide insertions or fusions, together with the genotype/phenotype linkage provided by coat protein’s ability to encapsidate its own mRNA, enabling two complementary approaches to vaccine design. First, the MS2 platform allows the deliberate, engineered presentation of already-identified epitopes. Second, it supports epitope identification by affinity-selection from complex random-sequence or antigen-fragment libraries by biopanning on antibody targets. When a selecting antibody has neutralizing activity, the VLPs it selects sometimes have the ability to elicit neutralizing antibody responses. The platform thus integrates epitope discovery and antigen presentation functions into a single particle.

## 3. Engineered Display of a Known Epitope: An MS2 VLP-Based Universal HPV Vaccine

Human papillomavirus (HPV) is the cause of nearly all cervical cancer. Existing HPV vaccines are based on genome-less VLPs assembled from the HPV L1 protein, the major structural component of the HPV virion. Like VLPs generally, L1-derived VLPs are highly immunogenic and therefore are extremely effective in preventing HPV infection [15]. However, because of L1 variability, over a hundred different HPV types exist in nature. Since the efficacy of existing HPV vaccines is largely restricted to homologous serotypes, VLPs made from HPV16 L1, for example, protect against HPV16 infection but are largely ineffective against other types. This is why the most broadly protective vaccines presently comprise nine different type-specific VLPs, which together protect against the most common HPV infections. The minor capsid protein, L2, is more conserved than L1, but is not present in these vaccines, and in any case is normally hidden and therefore non-immunogenic in the authentic HPV virion. L2 is apparently only transiently exposed during cell entry, at which point it becomes susceptible to neutralization by anti-L2 antibodies if, somehow, they are already present. We produced two versions of a universal HPV vaccine by introducing a 17-amino acid peptide from L2 at the N-terminus of MS2 coat protein, and into the AB-loop of PP7 coat protein. We immunized mice with the resulting VLPs. In both cases, animals mounted high titer anti-L2 responses that conferred protection against genital infection by highly divergent HPV pseudovirion serotypes. Importantly, the VLP-induced antibody responses were durable, falling only a slightly during sixteen months after immunization. [16,17,18].

## 4. Vaccines by Affinity-Selection

In addition to its role as the major structural protein of the virus capsid, coat protein functions as a specific RNA-binding protein to accomplish translational repression of replicase synthesis and encapsidation of the viral genome. Both functions depend on recognition of a hairpin at the start of the replicase cistron. As coat protein accumulates during the late phases of infection the dimer recognizes this hairpin (the translational operator) and prevents access of ribosomes to the replicase translation initiation site. This RNA-binding event was also thought to serve as a unique packaging signal (or *pac* site) responsible for genome encapsidation. In this view, a single RNA-protein interaction mediated the transition from the viral genome synthesis phase to the virus assembly stage of the infection cycle. We now understand that this picture is too simple. In reality, genome encapsidation is accomplished through interactions of multiple coat protein dimers with operator-like structures distributed at more-or-less regular intervals throughout the viral RNA [19]. The coat protein-operator/pac site interaction itself is the strongest of these interactions but is not itself entirely indispensable. Phage viability persists even when the coat protein-*pac* site interaction is completely inactivated by mutations in the operator hairpin [20]. Such mutants make smaller plaques, but they are not dead, presumably because the packaging function is redundantly distributed throughout the genome. This may account for the fact that coat protein expressed from a plasmid efficiently encapsidates its own mRNA even in the absence of its unique translational operator/pac site [11].

This ability to package its own RNA accounts for the linkage of genotype to phenotype that makes affinity-selection on the MS2 platform possible. The ~400 nucleotides of the coat sequence itself are fully capable of efficient packaging into VLPs. Complex peptide libraries, whether random sequences or antigen fragments, can be produced by insertion into the AB-loop. The resulting VLPs encapsidate the same mRNA that encodes their coat protein and whatever specific guest peptide it displays. Libraries are then subjected to biopanning on an antibody target followed by reverse-transcription and polymerase chain reaction to recover affinity-selected sequences. When recloned, the sequences produce VLPs for additional rounds of biopanning. Usually, two to four rounds are sufficient to arrive at a relatively simple, or even unique population of affinity-selected VLPs that bind a target antibody. Depending on the complexity of the target epitope, the resulting peptide can be an efficient epitope mimic, able to elicit antibodies with specificities like that of the selecting antibody. In other words, a neutralizing antibody can select a vaccine that elicits a neutralizing antibody response. The approach is most likely to succeed when the epitope is linear. Conformational epitopes are harder to mimic with peptides. Below we present an example.

## 5. Affinity-Selection from Random-Sequence Libraries

The malaria blood stage is a potential vaccine target. Most disease pathology comes from parasite multiplication within red blood cells, and natural immunity, when it exists, seems to depend largely on antibody responses to blood stage antigens. The main barrier to a malaria vaccine is the identification of an antigen able to provoke a strong immune response that neutralizes a wide range of parasite variants. An ideal vaccine antigen would be conserved across a broad spectrum of *P. falciparum* strains and would be essential for parasite viability. The merozoite protein called RH5 meets these criteria; it is necessary for parasite invasion of erythrocytes and its amino acid sequence is conserved across a wide range of *P. falciparum* strains. The essential role of RH5 is affirmed by its presence in all strains tested so far, and by the complete failure of efforts to genetically delete it. RH5 is exposed only transiently during cell attachment and entry, a fact that likely explains its poor immunogenicity. Although it is only briefly exposed to the immune system, it is nevertheless susceptible to neutralization when antibodies are already present at the time the pathogen attempts entry. Only a minority of patients produces a significant anti-RH5 response, and then only after prolonged chronic malaria exposure.

Anti-RH5 antibodies, including certain mAbs potently inhibit invasion. We conducted affinity selection using one such antibody, 5A08, hoping that the resulting VLP selectant would elicit antibodies that recognize the target epitope on RH5 itself and inhibit entry. These experiments used a mixture of four MS2-based random-sequence peptide libraries comprised of 6-mers, 7-mers, 8-mers and 10-mers, each with ~10^10^ individual members. After only two selection rounds, all of the several dozen selectants we analyzed had converged on the same peptide sequence, SAIKKPVT [21]. Comparison of the peptide to the RH5 sequence reveals a four-amino acid identity (AIKK) near the N-terminus, apparently identifying the 5A08 epitope. Immunization of mice with the 5A8 VLP selectant yielded antisera that reacted with RH5 in schizonts and with the purified protein in ELISA, and inhibited invasion of red blood cells.

## 6. Antigen-Fragment Libraries

Advances in DNA synthesis technology have given us the ability to conveniently produce libraries representing diverse fragments of any chosen antigen. Microchip-based methods enable the programmed parallel synthesis of many thousands of specific oligonucleotide sequences, which can then be used as mutagenic primers to introduce AB-loop insertions into MS2 coat protein. For example, we made VLP libraries designed to theoretically display all possible 10-mer peptides of the dengue virus 3 proteome [22]. Dengue virus proteins are made from a single large polyprotein by proteolytic processing. To construct the library, we scanned through the polyprotein sequence with a 10-amino acid window in 1-amino acid steps. Reverse translation yielded DNA sequences encoding each polyprotein 10-mer. Primer design was completed by attaching flanking sequences able to anneal to the site of insertion in MS2. After synthesis on a microchip, the mixture (~4000 sequences) was amplified by PCR to obtain an amount sufficient to serve as primers in a site-directed mutagenesis reaction [23] to create the library. We introduced the plasmid library into an *E. coli* expression strain to obtain the actual VLP library, which was then subjected on biopanning on dengue convalescent patient sera. The selected population was analyzed by Ion Torrent sequence analysis, allowing creation of a profile representing the various linear epitopes recognized by antibodies in infected individuals. We have extended the method to other viral and bacterial pathogens, including Zika virus (unpublished) and Chlamydia trachomatis [24].

**Figure 2:**
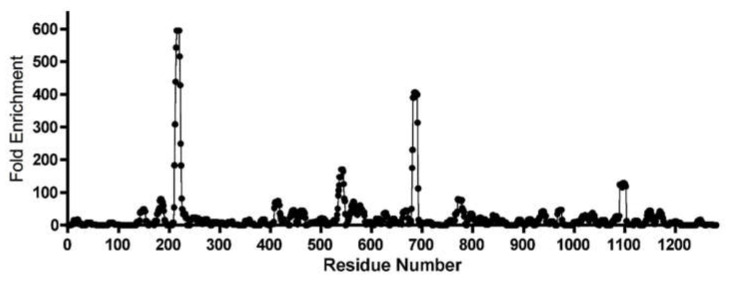
One example of affinity-selection of epitopes from a SARS-CoV-2 antigen fragment library. Peptide-VLPs were enriched by affinity-selection on serum with high neutralization activity from a single patient. Individual peptides were identified and their relative abundances relative to non-neutralizing sera were determined by Ion Torrent sequence analysis. Their positions in the spike protein amino acid sequence (x-axis) are plotted against their relative enrichment (y-axis). Similar plots were obtained for spike and other viral structural proteins and with serum from a number of patients (not shown). Those results will be joined with these as part of a fuller analysis to be presented elsewhere.

**Figure 3:**
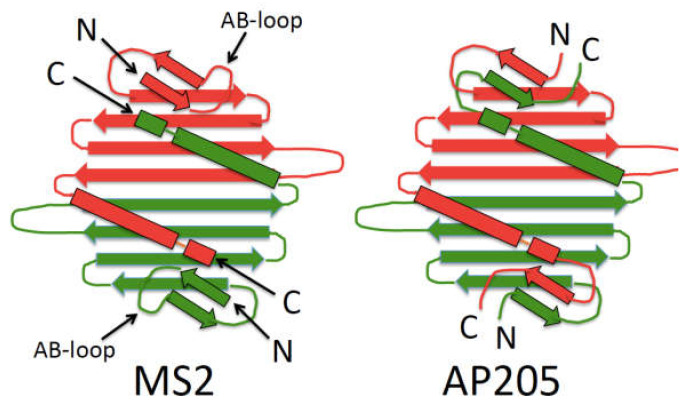
A comparison of the structures of MS2 and AP205 coat proteins. Despite extensive divergence of amino acid sequence, their three-dimensional structures are largely conserved. Note however, that the structural homolog of the N-terminal-most ß-strand of MS2 is moved to the C-terminus in AP205, causing the two sequences to be related by circular permutation.

Another illustrative example comes from ongoing unpublished work that seeks to identify linear epitopes recognized by antibodies in the sera of SARS-CoV-2-infected patients. In this case, an antigen fragment library was constructed on MS2 VLPs displaying a complex mixture of peptides ranging from 9 to 14 amino acids in length. They scan through the sequences of each of four viral structural proteins, namely spike, nucleocapsid, envelope and membrane proteins in steps of three-amino acids. Patient sera were chosen for their high neutralizing activity and applied to a single round of biopanning. The abundance of each peptide in the selected VLP population was determined by Ion Torrent sequence analysis, and then its relative enrichment was determined by normalizing it to its abundance after selection by non-neutralizing serum, which we then plotted against its position in the amino acid sequence (Figure 2). A typical result for the spike epitopes recognized by one patient’s sera is shown in Figure 3. VLPs from each peak will be tested for the ability to elicit neutralizing antibodies in animals.

## 7. PP7 VLPs

Bacteriophage PP7, a virus of *P. aeruginosa*, produces a coat protein that diverges markedly in amino acid sequence from MS2. Nevertheless, it folds into a highly similar dimer that also assembles into an icosahedral VLP when expressed from a plasmid in *E. coli*. As noted briefly above, we previously produced one version of a universal HPV vaccine based on insertion of an L2 epitope into one of PP7′s AB-loops [25]. Like MS2, PP7 coat protein’s tolerance of AB-loop insertions benefits significantly from the single-chain dimer construct. Although we have not yet specifically utilized PP7 VLPs for affinity-selection, the observation that they encapsidate coat-specific mRNA [25] suggests that, like MS2, they could be used for epitope identification by biopanning on antibody targets.

Zhao et al. have more recently taken PP7 VLP engineering to another level by demonstrating its tolerance of fusions at both the N- and C-termini [26]. Peptides, and even protein domains as large as 150 amino acids, are frequently tolerated. They also showed that peptides can be displayed by insertion into the linker that joins the two halves of a PP7 single-chain dimer. By utilizing this junctional site and the C-terminus together, a peptide epitope and a protein domain were displayed on the same VLP. Surprisingly, single-chain dimer recombinants assembled into T = 4 icosahedra. Given one display site per single-chain dimer, these particles displayed 120 copies of the foreign protein rather that the 90 expected of a similar T = 3 particle or the 180 obtained from assembly of the conventional coat protein dimer. These observations raise the possibility that PP7 could serve as a more generalizable display platform than MS2, which in its current configurations at least, does not so easily tolerate extensive fusions at its termini. Less coat protein crowding at the three-fold symmetry axes of PP7 presumably explains this difference; the PP7 structure is simply more open at these sites. It also has the added advantage of high thermal stability conferred by the presence of inter-dimer disulfide crosslinks. PP7, therefore, merits additional investigation as a versatile display platform.

## 8. Genetic Display on Qß VLPs

It has long been known that Qß virions contain two versions of coat protein. The major form is 133 amino acids in length and is structurally homologous to the MS2 structure shown in Figure 1. However, the virus particle also contains small amounts of the so-called readthrough protein, a C-terminally elongated form produced by partial suppression of an opal termination codon at the end of the coat reading frame. Readthrough protein is necessary for virus infectivity, but is not strictly needed for VLP formation, naturally suggesting its use as a display site for foreign peptides and proteins. Pumpens et al. exploited this property to perform some of the earliest experiments in peptide display on RNA phage VLPs [27,28,29,30]. In authentic phage, readthrough protein is normally present at only a few copies per virion, but the use of plasmids allows straight-forward manipulation of the two factors that control the level of its incorporation in VLPs: (1) the relative expression levels of the two proteins, and (2) the length of the extension. The maximum copy number for full-length readthrough under these conditions is around 10 per particle, whereas a version truncated to only 10–24 amino acids can be displayed in as many as 80 copies [28]. Foreign sequences inserted in place of the natural extension also end up on the VLP surface. Although peptide display valency can be varied over a wide range, again there is a limit to how many copies VLP assembly will tolerate. Early studies with recombinant proteins showed incorporation frequencies between 14% and 48% [30]. Later work by Brown et al. [31] fused the 58 amino-acid Z-domain of *S. aureus* protein-A to the Qß coat C-terminus and showed that, depending on growth conditions, between 20 and 30 copies could be presented on these mosaic VLPs.

In some cases, it has been possible to exploit the Qß readthrough phenomenon to actually display peptides and proteins on intact, infectious recombinant bacteriophages [32,33]. Apparently, some portions of the readthrough protein are dispensable and can be interrupted with foreign sequences. The importance of readthrough protein for infectivity and the existence of the virion’s variable upper limit on extension copy number imposes additional constraints on display density that may restrict the utility of infectious Qß, but the ability to produce the particle by growing it as a self-replicating viral entity introduces the possibility of using its relatively low replication fidelity to spontaneously mutagenize and then affinity-optimize displayed peptides and proteins [32,33]. So far, Qß has been little used for this purpose, but it deserves further effort.

## 9. Display on Qß VLPs by Chemical Conjugation

Genetic insertion of sequences into coat protein genes is not the only approach to peptide display. Chemical conjugation of chemically synthesized peptides has long been used to produce highly immunogenic epitope-specific vaccines. It has the important advantage that because the technique utilizes preformed VLPs, there is no worry that the added peptide will interfere with coat protein folding and VLP assembly. Except in cases where the peptide causes VLP aggregation (e.g., if it is too hydrophobic), production of the peptide-VLP vaccine is virtually assured. Furthermore, peptides can be conjugated at even higher valencies than are typical attainable by genetic display. This, in turn, can sometimes give even higher immunogenicity. A variety of conjugation chemistries are available, but one commonly-used approach simply incorporates a cysteine residue at one end of the synthetic peptide, and then employs a bifunctional cross-linker (e.g., succinimidyl 6-[(β-maleimidopropionamido) hexanoate, SMPH) to join it to lysine amino groups on the VLP surface. Conjugation efficiency depends on the availability of primary amines, of course, a property that varies among the various RNA phage coat proteins. Each Qß subunit provides up to six available surface lysines and has proven itself a suitable target for such reactions. The VLP of the *Acinetobacter* phage, AP205 (see below), is similarly reactive, as are mutant MS2 VLPs engineered to present additional lysines (unpublished results). VLPs displaying an average of 240 peptides or more are routinely obtained.

Epitopes displayed at these densities provoke high-titer anti-peptide responses almost invariably. In fact, peptide-VLPs are so immunogenic that they can efficiently break immune tolerance and elicit high-titer antibody responses even against self-antigens [34,35,36,37,38]. This has led to the idea that it could be possible to replace expensive, inconvenient-to-administer therapeutic monoclonal antibodies with cheap, and potentially longer-lasting vaccines. Although perhaps suitable for only a subset of diseases now treated with mAbs, such vaccines could be effective alternatives in some cases. Peptide-VLP vaccines have been produced for a variety of self-antigens. For illustration, consider the recent example of PCSK9 [39,40].

Proprotein convertase subtilisin/kexin type 9 (PCSK9) is a serum protein involved in the control of circulating low-density lipoprotein (LDL) levels. It functions by mediating the internalization and degradation of the LDL-receptor (LDL-R). Lowering LDL-R in turn leads to increased levels of circulating LDL-cholesterol. It has long been recognized that reduced PCSK9 activity leads to decreased serum cholesterol levels and to decreased risk of heart disease. So, for example, loss-of-function mutations of PCSK9 lead to dramatically decreased circulating cholesterol and reduced risk of heart disease. Gain-of-function mutations have the opposite effect. One approach to PCSK9 inhibition is to elicit an anti-PCSK9 antibody response with a vaccine capable of breaking tolerance to this particular self-antigen. Crossey et al. describe Qß VLPs displaying synthetic peptide epitopes from the PCSK9 sequence [39,40]. They have the predicted effects on LDLR and cholesterol levels in immunized animals.

Many potential vaccine targets are non-peptide/protein antigens, of course. Alternative conjugation chemistries make possible the attachment of huge variety of molecular types to VLPs. Yin et al., for example, [41,42,43] used click chemistry to attach tumor-associated carbohydrate antigens to Qß’s amino groups. Carbohydrate antigens require conjugation to protein carriers simply for the T-help needed for durable IgG antibody responses, of course. However, in addition to serving this carrier function, VLPs have the added advantage that they present dense, repetitive antigen arrays for potent B-cell stimulation. Display valency is an important variable influencing the strength and quality of antibody responses to carbohydrate antigens and by adjusting the reaction conditions, a wide variety of antigen densities are achievable.

Finally, Qß conjugants have also been used to raise antibodies even to some small molecule haptens, e.g., nicotine. In that case, the goal was to produce an anti-smoking vaccine. Although clinical trials failed to demonstrate the desired level of anti-smoking effect [44], the vaccine was nonetheless effective in provoking high titer antibodies [45,46], The hope persists that VLPs might yet provide the basis of vaccines against drugs of abuse.

## 10. AP205 Offers New Display Modes

In references [47,48,49], *Acinetobacter* phage 205 (AP205) is another of the single-strand RNA bacteriophages, but the sequence of its coat protein diverges so sharply from MS2’s that it is difficult to see any sequence similarity. It is in the comparison of their homologous 3D structures that a clear relationship is revealed. Their tertiary structures are obviously similar, but their sequences are, in effect, circular permutants of one another [50]. Compared to MS2, it is as though a segment of roughly 10 amino acids had been transposed from the AP205 N-terminus to its C-terminus. In MS2, the AB-loop occupies amino acids 13–15, so the AP205 circular permutation effectively moves its N- and C-termini to the top of what would be the AB-loop in MS2 (and most other RNA phages) (Figure 3). This means that the coat protein termini, which are both relatively inaccessible in the MS2 VLP, present themselves prominently at the AP205 VLP surface. Peptides fused to either end are not only thoroughly exposed, but also positioned so they are unlikely to interfere with assembly.

Tissot et al. took advantage of AP205’s structural peculiarities to display a selection of peptides and small proteins ranging in size from 8 to 55 amino acids [47]. Linker/spacer sequences were attached genetically to the coat protein ends together with appropriate restriction endonuclease cleavage sites to facilitate recombinant manipulation. VLPs were obtained for each of the six peptides tested. Since then, additional experience shows that AP205 is not quite the universal acceptor of fusions that was originally hoped for (unpublished results), but is nevertheless well-suited to the N- and C-terminal presentation modes. In a more recent illustrative example, Liu et al. fused the receptor-binding domain of the SARS-CoV-2 spike protein to the C-terminus of an AP205 coat protein single-chain dimer [51]. Although produced in large amounts in *E. coli*, the protein failed to fold properly, and instead accumulated as insoluble aggregates. Nevertheless, the protein was refolded from these inclusion bodies and assembled in vitro into a VLP with the expected immunogenicity. In mice it elicited antibodies that bind the spike protein and neutralize the virus.

AP205 has also shown itself an effective platform for the so-called spytag/spycatcher technology, an efficient cross-linking system based on the covalent bond forming capability of CnaB2, an immunoglobulin-like collagen adhesin domain from the fibronectin binding protein of *S. pyogenes*. CnaB2 naturally forms an autocatalyzed, intramolecular isopeptide linkage that stabilizes its fold. However, the domain can be split into 138- and 13-amino acid fragments, called spycatcher and spytag, respectively, that retain the ability to form this linkage even when each is fused to different proteins. Spytag/spycatcher therefore serves as a universal intermolecular crosslinking technology that requires no additional chemistry to covalently join two proteins. So, for example, fusing spytag to one of AP205 coat protein’s termini creates a VLP that can accept any other protein which has already been fused to its spycatcher partner [52,53]. Because the process attaches a protein to a preformed VLP, the necessity of genetically fusing the antigen directly to coat protein is eliminated, along with any tendency of the fusion to interfere with coat protein folding or assembly. It does, of course, require the separate production of platform and antigen with the necessity of performing a subsequent reaction that joins the two. Display density of large antigens can be limited by surface crowding considerations, but valencies of at least 60 copies/VLP can typically be obtained. Because it makes possible the relatively straightforward synthesis of VLPs displaying practically any arbitrarily chosen antigen, AP205 has become a popular plug-and-play vaccine platform [53,54,55,56,57,58]. Recently, Fougeroux et al. described the production of a promising vaccine candidate for SARS-CoV-2 by decoration of AP205 particles with the virus’ receptor-binding domain [53].

## 11. Even More RNA Phages

About a thousand new members of the ssRNA phage family were recently identified in metagenome sequence data. Genes for coat proteins of eighty of these never-cultured phages were synthesized, cloned and over-expressed as VLPs in bacteria [59]. Remarkably, the 3D structures of twenty-two such VLPs were determined by X-ray crystallography [60]. Even though their amino acid sequences diverge widely, each preserves certain key features of the tertiary fold exemplified by MS2. Each has at least a four-stranded ß-sheet (which becomes eight stranded in the dimer) and at least one alpha-helix extending over the ß-sheet of its companion subunit. The diversity of RNA phage VLPs of course offers new potential vehicles for peptide antigen presentation. Indeed, several have already shown they can accept both N- and C-terminal fusions [61]. Evolution has been exploring VLP design space for many millions of years. The diversity of these ancient forms, together with the potential of new protein engineering and directed evolution methods to improve their properties, makes us feel that RNA phage VLPs still have contributions to make.

## Data Availability

Data supporting any original work reported here is available from the corresponding author upon request.
